# 3 Months without the car in Bielefeld, Germany– a mixed-method study exploring individual motivation to participate in a municipal intervention

**DOI:** 10.1186/s12889-024-18266-7

**Published:** 2024-03-11

**Authors:** Anna Christina Nowak, Susanne Lopez Lumbi, Timothy Mc Call

**Affiliations:** 1 School of Public Health, Bielefeld University, 33615 Bielefeld, Germany; 2 Sustainable Environmental Health Sciences, Medical School OWL, Bielefeld University, 33615 Bielefeld, Germany; 3 Working group Environment and Health, School of Public Health, Bielefeld University, 33615 Bielefeld, Germany

**Keywords:** Behavior change, Mobility, Mixed-methods design, Pro-environmental behavior, Health, Well-being

## Abstract

**Background:**

Climate change is a major public health issue worldwide. To achieve climate targets and reduce morbidity, a paradigm shift in individual behavior e.g., in mobility, is needed. Municipal interventions can motivate individuals to engage in climate-friendly behavior through different psychological mechanisms. In order for successful interventions, it is necessary to gain better insight from study participants and their reasons for participating in mobility projects (e.g., motivational aspects).

**Materials and methods:**

A mixed-methods design was used to evaluate reasons and characteristics of people for participating in an municipal mobility intervention. The quantitative sub-study assesses socioeconomic characteristics, environmental awareness and perceived stress. The qualitative sub-study explores motivation for participation and change, perspectives on car replacement and reasons for car use.

**Results:**

Results show that participants (*n* = 42) are rather high educated and show medium environmental awareness. Participants of the qualitative study part (*n* = 15) were motiviated to reduce car use already before the intervention and used the intervention as starting point or trial phase.

**Conclusions:**

Urban intervention projects with fitted recruitment strategies and better insights from study participants with the aim to motivate individuals to engage in climate-friendly behavior can help to strengthen sustainability and public health.

**Supplementary Information:**

The online version contains supplementary material available at 10.1186/s12889-024-18266-7.

## Introduction

Climate change is a major public health issue [1]. Especially, high-income countries contribute to climate change with high greenhouse gas emissions caused by resource-intensive lifestyles [2]. The consequences of climate change, like the increase in heat waves, droughts, and floods caused by heavy rainfall, may also have a significant impact on the health of the population in Germany [3]. Common health impacts include heat stress, allergic reactions, respiratory diseases, or injuries and deaths during extreme weather events, which also directly affect the health care system (ibid.). In order to mitigate climate change, a transformation on the individual, social, and structural level is necessary. Wynes and Nicholas [4] argue that high-impact actions, like avoiding air travel, eating plant-based diets, and living without a car, may contribute significantly to the Paris Agreement’s goal of limiting the global temperature rise to 2° C, but remain underaddressed by governments and education systems.

Even though the negative impact of motorized transportation on health and urban areas is well known [5, 6], the CO_2_-emissions caused by road vehicles in Europe remain high [7]. In particular in high-income societies the car continues to be of great importance [8]. It is estimated that cars account for 59.4% of the road traffic CO_2_-emissions in Europe [9]. In Germany 80% of greenhouse gas emissions from all transportation modes are due to motorized individual transport (cars and two-wheeled vehicles) [10]. Additionally, the traffic-related ambient noise and air pollution promote health impacts, like cardiovascular diseases, sleep disorders, and depression [11]. Therefore, to achieve climate targets, as well as reduced morbidity, a paradigm shift in motorized individual transport is needed that includes not only technological changes and sustainable planning and infrastructure, but also individual behavioral changes [12, 13].

Interventions can motivate individuals to engage in climate-friendly behavior through different mechanisms based on pychological behavior models. The use of information about pro-environmental behavior is one such mechanism. In addition to showing the consequences of environmentally relevant behavior [14], instructions on how to carry out behavior [15] and information on the behavior of others [16] are also information-specific measures that can promote climate-friendly or pro-environmental behavior. Financial incentives are another important tool to encourage pro-environmental behavior. Money, vouchers, and discounts have been shown to encourage pro-environmental behavior [17]. Consequently, financial incentives to avoid car use, for example, could reduce air pollution on a local level and simultaneously reduce greenhouse gas emissions on the global level. In parallel, increasing physical activity through alternative mobility like cycling or walking, has the potential to positively influence individuals’ health [18].

Information provision and financial incentives are only some of the mechanisms used in environmental psychology and public health interventions, however. In a meta-analysis the effectiveness of different behavior change methods were assessed. Using cognitive dissonance, goal setting, prompts, and social modelling were the most effective treatments with the highest effect sizes. It was shown that across methods 69% of people in the treatment groups were more likely to adapt pro-environmental behavior than the people in the control groups. Changes in car use, however, were not assessed in any of the included studies [19]. Pro-environmental behavior is further associated with nature connectedness [20, 21] and environmental awareness [22]. Results of a meta-analysis showed that a deeper connection to nature may partially explain why some people have a greater engagement in pro-environmental behavior than others [20].

In this paper, we analyzed the characteristics of people who are willing to change to more sustainable mobility behavior during an intervention and their reasons for participating. The assessed intervention was planned and conducted by the Environmental Office of the German city Bielefeld and was titled “3 months without the car” (German original title: “3 Monate ohne Auto”). The intervention encouraged interested citizens of Bielefeld to leave their car and use more climate-friendly alternatives for three months through financial incentives, group meetings, information events, and feedback provision. The quantitative characteristics and environmental values of participantes were complemented by the qualitative statements with the aim to aswer the following research questions:

What are the characteristics and environmental values of people who voluntarily participated in the intervention “3 months without a car” in Bielefeld, Germany?

What are participants’ reasons for car use and the motivation for participating in a project reducing car use?

## Materials and methods

### Study Design

To evaluate the intervention “3 months without the car” we chose a mixed-methods design. We used a convergent parallel design [23] to illuminate different aspects of our research questions. The quantitative results are used to illustrate the characteristics of the study population. The qualitative results serve to explore the reasons for participation in the intervention and perspectives on mobility in general. The Environmental Office of Bielefeld launched a call for participation in the local press, the communal website, and their social-media channels. Interested citizens needed to apply proactively. All participants received a link to an online questionnaire via the project leader of the Environmental Office and a reminder after one week. All participants of the quantitative survey were invited to take part in the qualitative interviews. To do so, they could provide their contact information in the online survey, in accordance with data protection regulations. Overall, *n* = 23 participants provided their e-mail adress for the qualitative part of the study. In order to protect participants’ anonymity, the quantitative and qualitative data were not linked. However, all participants in the qualitative interviews took part in the quantitative survey. Due to the missing link of the data the merging process is rather general and results will be compared only in the Discussion section.

**Fig. 1:**
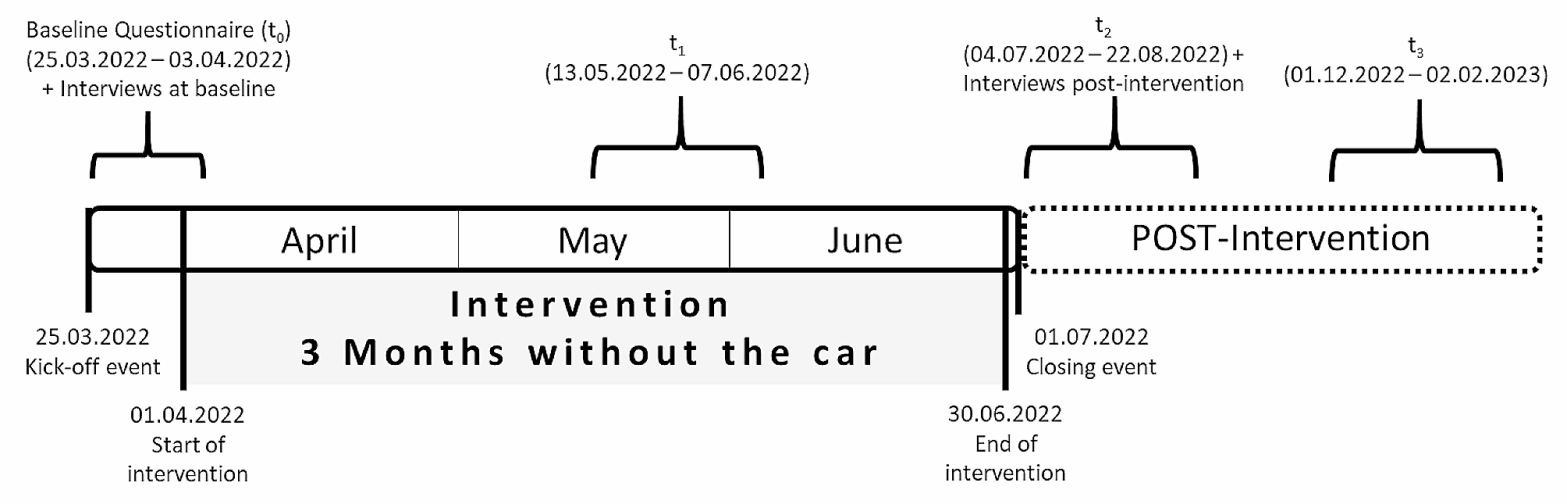
Timeline of the municipal intervention “3 Months without the car”

The study design included four quantitative assessment points (baseline, after 1.5 months, at the end of the project, and 6 months after the project) and two qualitative assessment points (at the beginning of the intervention and at the end of the intervention) (Fig. 1). The results presented here, however, are from the baseline survey and the qualitative assesments from the beginning of the intervention.

### Intervention

The intervention initiated by the Climate Council of Bielefeld, required participants to leave their cars for three months (April 2022 to June 2022) and use alternative means of transportation (https://www.bielefeld.de/autofrei). The statement of relinquishment to not use the car was contractually agreed at the beginning of the project. The only exception was emergencies. In the first meeting regional companies that offer alternative transportation presented their products. Some companies offered participants special conditions for the duration of the project (e.g., no basic fee for car sharing or trial subscriptions for public transport).

For additional expenses, like public transportation tickets, cargo bike rental fees, or bicycle equipment costs, participants received a remuneration of up to 400€. All 50 applicants joined the intervention group. Due to SARS-CoV-2-pandemic regulations the group met online during the intervention to exchange experiences.

### Quantitative Procedure and Measurement

For the quantitative assessment a German language questionnaire was created (supplementary file 1). First, participants were asked about their mobility habits (e.g., number of trips on a specific day, transportation mode used, and satisfaction with different transportation modes) with questions adapted from Engel and Pötschke [24]. The second section included questions about the participants’ health and well-being via different validated tools (i.e., the WHO-5 and the Perceived Stress Scale) [25, 26]. Additionally, the environmental awareness of participants was assessed with a validated tool of the German Federal Environmental Agency [27] and nature connectedness with the Extended Inclusion of Nature in Self scale (INS) [28]. Finally, participants were asked about their demographic characteristics (e.g., age, sex, and education).

#### WHO-5 & general health

The WHO-5 is a quick screening tool for well-being in regard to the last two weeks. It consists of five 6-point Likert scale items (0 = “at no time” to 5 = “all of the time”). The scale items are derived from depression, anxiety, general health, and general well-being questionnaires [29] and show a high overall reliability in a cross-country sample [29]. Cronbach’s alpha for this sample is 0.77. Additionally, participants were asked to assess their general health (very good, good, intermediate, bad, and very bad).

#### Perceived stress scale (PSS-10)

The Perceived Stress Scale is a tool originally developed by Cohen et al. [30] and measures perceived stress with 10 5-point Likert scale items (1 = “never” to 5 = “very often”). The scale reflects the dimensions of helplessness and self-efficacy and refers to the last month [26]. The German version was validated with a high internal consistency [26]. The Cronbach’s alpha in this sample is 0.78.

#### Inclusion of Nature in Self scale (INS)

**Fig. 2:**
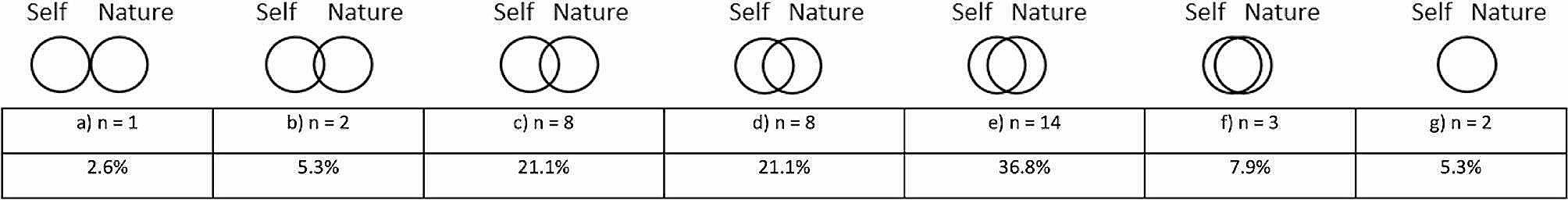
Distribution on the inclusion of nature in self scale [33] (*n* = 38)

The INS is a visual analogue scale where participants choose a picture which represents their relationship with nature most. The scale shows a series of seven overlapping circles with the labels “nature” and “self” [31, see Fig. 2]. The scale ranges from complete separation (0) to a perfect match (6) between “self” and “nature” with the degree of overlap symbolized by circles. According to Schultz [31], the test instrument is reliable and valid for the operationalization of nature connectedness.

#### Environmental awareness

The German Federal Environmental Agency has been evaluating the environmental awareness in the German population for over 20 years with a designated tool that has been validated and is continuously revised [27]. The tool measures the three components affect, cognition, and behavior. The scale has 23 predominantly 5-point Likert scale items. The components affect (7 items) and cognition (8 items) represent statements that one agrees or disagrees on (1 = “strongly disagree” to 5 = “strongly agree”). All but one item of the behavior component (7 items) reflect on the frequency of behaviors via 5-point Likert scales (1 “never” to 5 “always”). One dichotomous item concerns the purchase of electricity (“My household purchases conventional OR green electricity.”). The scales are standardized into scales from 0 to 10 for comparability.

#### Quantitative analysis

The quantitative data is presented descriptively. Data cleaning and analysis was conducted with R Statistical Environment [32] including the ‘tidyverse’-package [33]. The items from the validated tools were analyzed according to their description. The WHO-5 is a sum scale. Answers have points from 0 to 5 and values are summed up, resulting in raw values from 0 to 25. Higher values represent higher quality of life and well-being. The multiplication of the raw values by four results in values from 0 to 100 for presentation in percentages [25].

The PSS-10 is also analyzed via sum scores. The helplessness sub-scale consists of six items (resulting in scores from 6 to 30). The self-efficacy sub-scale consists of four items (resulting in scores from 4 to 20). The total score is computed with the sums of the helplessness-scale and the reversed self-efficacy scale (scores range from 10 to 50). Higher values represent higher perceived stress [26].

Environmental awareness is presented via means with possible values ranging from 0 “not aware at all” to 10 “strongly aware”. Five items need recoding as they are inverse. The means of the components (i.e., affect, cognition, and behavior) are also calculated.

### Qualitative Procedure

The convenience sample was formed by contacting all participants who left their e-mail adress (*n* = 23) for further contact. *N* = 15 people agreed to participate in the qualitative study. It can be assumed that data saturation was reached because we included a homogenous study population [34] but no empirical approach to assess saturation was implemented. Written informed consent was obtained before the interview appointments. Interviews were conducted online via an online video conference tool (i.e., Zoom), by phone, or in person at the participants’ home, depending on the participants’ preferences. The interviews were conducted by a female interviewer (ACN, PhD, postdoctoral researcher) with experience in qualitative research. To explore the reasons and motivation for giving up car use and participating in the project, we conducted qualitative semi-structured interviews [35]. Except for a couple who opted to be interviewed at the same time, the interviews were conducted individually. The interview guide developed by the project team contained open questions exploring: (1) daily trips, daily activities, and means of transport; (2) previous experiences with car alternatives; and (3) motivation to participate in the project (supplementary file 2).

#### Qualitative analysis

All interviews were audio-recorded and transcribed verbatim. For time reasons, transcripts were not returned to the interviewees. In addition, field notes were taken to record special observations during the interviews. Interviews were analyzed using a content-analysis approach [36]. First, deductive main categories were developed based on the interview guide. Then, the main categories were inductively differentiated into subcategories. The main- and subcategories were defined by the author ACN. The final category system was tested by all authors on five interview transcripts; divergences were discussed and resolved. The analysis of the transcripts has been conducted with MAXQDA 2022 [37].

## Results

### Quantitative analysis

#### Demographic characteristics

Demographic data was available for *n* = 42 participants. The mean age was 41.8 years (range: 23–74 years). Most of the participants identified as female (*n* = 28, 66.7%). The education level distribution was skewed with a high percentage of participants with higher education: 64.3% (*n* = 27) of particpants completing university degrees, 19.0% (*n* = 8) upper secondary education, and 16.7% (*n* = 7) lower secondary education. The household size ranged from 1 to 5 persons with a mean household size of 2.7 (SD = 1.5). Households with one (*n* = 13, 31.0%) or four persons (*n* = 14, 33.3%) were most common. Of the participants 40.5% (*n* = 17) were living with children younger than 12 years old. More than 65% of participants lived in districts that are central or close to the city center.

#### Mobility behavior

The data for mobility behavior was available for *n* = 37 participants. Most of the participants used a car for their daily routes (*n* = 35, 94.6%). In addition to the car, bicycles or e-bikes were primarily used (*n* = 30, 81.1%). Regarding public transport, *n* = 14 participants (37.8%) used the bus and *n* = 18 participants (48.6%) the tram for daily routes.

#### Health status

Generally, participants (*n* = 38) perceived their health to be either good (*n* = 19, 50.0%) or intermediate (*n* = 15, 39.5%). Fewer participants perceived their health status to be very good (*n* = 4, 10.5%) and nobody indicated a bad or very bad perceived health status. The WHO-5 showed an average score of 58.8 points (SD = 16.3, range: 16–84) regarding well-being during the last two weeks before the project.

#### Perceived stress

Regarding the month before the project, participants (*n* = 33) rated themselves with an average of 17.7 points (SD = 3.2, range: 10–26) on the helplessness-scale. Self-efficacy was rated quite high with a mean score of 14.3 (SD = 1.9, range: 11–18). The overall perceived stress was rated with a mean score of 27.4 (SD = 4.7, range: 16 to 39).

#### Nature connectedness and environmental awareness

Most of the participants (*n* = 14, 36.8%) rated their nature connectedness with option e) on the Inclusion of Nature in Self scale (Fig. 2), meaning that they saw themselves quite connected with nature but did not see themselves as one with it. The overall distribution was centered to the options in the middle.

With a mean score of 5.94 (SD = 0.63, range: 4.78–7.17) the average environmental awareness of the participants (*n* = 28) indicated neither high nor low awareness. The sub-scales (i.e., affect, cognition, and behavior) indicated different tendencies. The affect-scale indicated an average score of 4.73 (SD = 0.78, range: 2.86–6.79), the cognition-scale an average score of 7.05 (SD = 0.52, range: 5.94–7.81), and the behavior-scale an average score of 5.90 (SD = 0.64, range: 4.67–6.96), respectively.

### Qualitative analysis

**Fig. 3:**
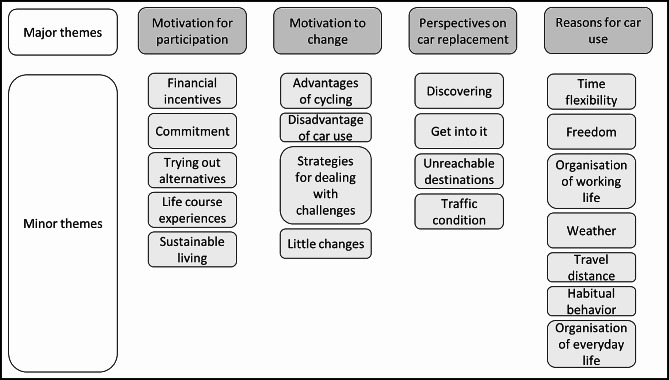
Major and minor themes of the coded interviews

In total, *n* = 16 persons participated in the interviews at baseline (t_0_), including 13 women and three men aged between 25 and 74 years old. Five interviews were conducted face-to-face at the participants’ homes, five interviews by telephone, and five interviews online with a video conference tool (e.g., Zoom). Nine of the participants lived with children and six of them lived alone. The interviews lasted about 30 min on average. Interviewees described several reasons for participating in the intervention, their motivation to change their mobility behavior, reasons for using a car in their daily lives, their perspectives on car replacement. Figure 3 gives an overview of the major and minor themes coded for the interviews. The results will be presented via the major themes. For further description and examples of quotes to all minor themes see supplementary file 3.

#### Motivation

Interviewees expressed their motivation in different ways. On the one hand they talked about the financial support of the intervention as well as advantages of alternative modes of transportation and the disadvantages of car use. The project gave the opportunity to try alternative forms of mobility and at the same time provided the impetus to live without a car. Many participants already had positive experiences with other forms of transport beforehand. Therefore, participants’ motivation was mainly to maintain or consolidate their decision to live without the car. On the other hand participants also reported about their general motivation to change their mode of transportation.

#### Motivation for participation

Motivation for participation refers to the inherent reasons for participation in the intervention. Many participants already wanted to get rid of their car before taking part in the project. At the same time, they experienced mental or structural challenges in this deciscion. One interviewee, for example, described her childhood experience in the “*flat countryside*” as having perceived the car as a “*world of mobility*” (I11, 41). Another interviewee described the alleged use of the car and the justification to himself as follows: *“I still have to go to the gym, then I can take the child with me. In the end, I was really just deceiving myself. […] To regain a clear conscience, right? That you don’t have to feel bad. That’s why I thought, come on, give it a try.*” (I9, 39). The formal commitment to not using cars as part of the project was therefore experienced as helpful to overcome “*one’s weaker self*” (I6, 74). The project structure invited participants to see whether long-term changes are even possible: “*I just wanted to see if I could overcome my weaker self and get out of this comfort zone*” (I9, 39).

Trying out alternatives and times without car was a high priority for many of the participants: “*And before I bought this car, I actually thought of maybe not having a car at all. But this is now the opportunity to try out whether you really want to do without it completely*” (I1, 29). On the one hand, this shows that participants were already thinking about living without a car before starting the intervention. On the other hand, the project was able to provide the impetus to tackle this new way of life.

Three interviewees highlighted the financial support from the intervention but also financial advantages due to a life without the car were mentioned. “*I hadn’t looked at things like tickets for public transport either, but if I were to do that, it would always be in addition to the car. And you don’t have to. So if you have a car outside the door, you don’t buy a ticket for public transport. And now that it’s being covered [by the intervention], so to speak, the inhibition threshold is simply lower*.” (I8, 49). The possibility of combining different transport options was also emphasised. The cost of ticket prices is becoming less relevant during the intervention. “*This start was stupid with the winter and then I travelled by train and thought it was so great that I was able to buy a bike ticket. I would never have bought that because I always think: ‘oah that’s so expensive it costs 3 euros and a ticket costs 4.80.’ Then I always think:'hey then I can only use it, so yes. Now you can buy a bike ticket'.”* (I13, 5). At the same time, the participants highlight that a realistic assessment of car use is obtained by participating. For many, the car is on the doorstep and has immense running costs, such as insurance and taxes. The high petrol prices are also a motivation to take part in the project. “*But if I have the car parked outside and then have to pay 50 euros for a train ticket, it’s always like, ‘hm, I’ve got the car parked here outside’. And now that it’s subsidised a bit, you get a realistic financial assessment of how much it actually costs me and isn’t the car that’s parked here on my doorstep actually more expensive*.” (I8, 47).

A part of the intervention were discounts from alternatives like car sharing and public transport which motivated participants to try new things. “*Exactly, so we’d have to get to grips with car sharing. Because, let me tell you, I’ve already signed up for it, because the registration is free thanks to this intervention, but I haven’t even borrowed a car yet.” (I6,39).*

Sustainability was mentioned by very few interviewees as the main reason for participation. Only one interviewee emphasised sustainability in order to “*set an example to the children that you can’t always get into the car quickly*” (I6, 78).

#### Motivation to change

Many interviewees had already changed their behaviour prior to the intervention and therefore had a high personal motivation in not using their cars. Seven people reported little change in their behavior as a result of taking part in the project. One interviewee, for example, described how the family uses the car only as “*exception*” (I3, 6). Seven interviewees discovered the bicycle as a suitable transportation mode before starting with the intervention, so they experienced little changes in their daily lives. In addition, structural and social success factors were also mentioned. For example, if alternatives are already available in the professional context, a car is no longer absolutely necessary in daily activities (I1, 45). Two interviewees describe the changes in their mobility behaviour as a result of the coronavirus pandemic: “*Well, coronavirus really was a rash, so I thought I didn’t want to be on the trains so much. I work in a hospice and then I thought, oh, that’s somehow too vulnerable for me. (…) and then I didn’t do any sport or go to the gym. I actually did some kind of sport three times a week. That was then cancelled. That probably came about through zoom, but it was all different somehow, it wasn’t me, I wasn’t at my limits, maybe it was something like that, or even stepping out of my comfort zone to see if I could move on my own. I think that was also a reason. To be able to get to my workplace myself without having to rely on anyone. Yes, and then that’s what I decided to bycicle”* (I13, 21). Exercise was also an important intrinsic factor for many other people to do without a car. Cycling was experienced as “*pleasant*” (I6, 75), “*relaxing*” (I4, 39), “*comfortable*” (I8, 33) and beneficial to health (I1, 79). The high flexibility and “*being outside*” were also emphasised.

Some interviewees described the switch to cycling as a challenge because they considered themselves “*fair-weather cyclists*” (e.g., I1, 70) but use public transports as a strategy when it is “*raining cats and dogs*” (e.g. I5, 28). Apart from weather conditions, the traffic conditions or a fear of cycling were especially challenging. The interviewees had already thought of solutions for these challenging conditions (e.g., driving longer routes with less traffic, wearing a helmet, use of well asphalted road instead of the potholed cycle paths). Others took the project as a test phase to get rid of their car permanently. The strategies for dealing with challenges in particular clearly demonstrate a high level of motivation to give up the car.

A large number of personal reasons for wanting a change in terms of mobility arose from negative experiences with car use in public spaces. Some interviewees reflected on car use in advance and realised that the car is not used much in everyday life. *“But actually, when I’m on holiday, it just stands around, always. And that was also the reason why I thought, somehow this sucks, I have to get rid of it in the long term. Because it’s just so annoying looking for a parking space here in the city”* (I8,5) pointed one interviewee out.

#### Perspectives on car replacement

Perspectives on car replacement includes aspects that participants already experienced in the beginning of the intervention or thoughts in advance to the intervention.

Almost all of the interviewees felt that they had more time after switching because they had to organise their everyday life better; they had fewer appointments and/or unnecessary appointments were cancelled. Experiences of deceleration were in particular experienced as valuable and were used as quality time for themselves or with relatives (I4, I6, I7, I9, I11, I13):

“*What I’m noticing more and more now is that when you’re in such a hamster wheel all day and with a child and with work and you have a thousand to-dos, but when you just have to wait for the bus, it’s incredibly decelerating, because that’s 10 minutes a day where you just can’t do anything*” (I11, 25).

For some respondents, the bicycle was the first choice of transport because it ensured a high degree of flexibility and spontaneity– similar to the car (I4, I5, I12, I13, I15). Cycling also helped to increase their health and well-being and to “*clear the mind*” (I4, 39). One interviewee described her experiences in and with nature because of her bicycle use in detail:

“*But I really like this morning when I set off here and […] I also have some kind of experiences, for example East Westphalian weather lights […] such greenish veils in the sky and I thought:'hey, I wouldn’t have seen that otherwise', or you somehow encounter animal worlds or a lot of fog where I think:'oh, I wouldn’t really like to drive through that' and yet you manage to do it and I think that’s great, so to grow a bit with nature*” (I13, 23).

Better structural conditions made the switch easier. For example, one interviewee reported that she no longer needed a car to get to work because her employer offers car sharing. Furthermore, a good connection to public transport and easily accessible and regularly available car sharing services were experienced as a relief. According to the narratives, people generally stayed in closer proximity of their homes without having a car. This was especially true for families with small children who did not have to cover large distances in everyday life.

These positive experiences can also lead to long-term changes in habits. One interviewee described the effect that regular cycling had already had before the project: “*I think it’s great to set off at 6.00 in the morning. That sounds kind of awful, but I don’t even think about it. I get dressed, the clothes are already here. They’re packed and then I drive off and I’m at work at about 10 past seven. I think that’s great, because then I have a great sunrise at the Castle.*” (I13, 5).

#### Reasons for Car use

The main reasons for car use centered around commuting to work and family obligations. Interviewees who lived alone reported more frequently using their car for leisure activities and habitual short trips. The organisation of everyday life played a special role for car use as short trips were also made by car, for example to take the children to leisure activities or to pick them up spontaneously from friends, to go grocery shopping or when bad weather was forecasted. Above all, the car provided a high degree of flexibility in the organisation of everyday life, especially if the daily routine was planned in advance (I4). Habits played an important role in car use as well and were critically reflected upon by the participants. The participants’ critically questioned short distance car use, even described it as “*lazy ways*” (I1,37) that could easily be replaced by other means of transport or walking.

At the same time, living without a car meant that certain activities could no longer be implemented in their everyday life because the organisation became too complex (I4, I5, I11).

There are some places that are “*in the pampas*” (I14, 3). This meant that certain (leisure and working) activities could only be carried out by using a car or when accepting long journeys on foot or by bike. This also affected the care for older relatives:

*“So my biggest problem is simply that my mother lives in [neighbouring town] and I do visit her once a week, otherwise she doesn’t get out anymore, so to say, and I’m her reference who puts her in the car and drives with her to […] the plum blossom and also only such short distances, but at least that she sees something else.” (I13, 3)*.

Some reasons for car use also originated in the disadvantages of public transport. Frequent difficulties were the limited working hours of public transport– especially in the evenings (and on weekends), no connection between city districts– so that detours had to be accepted, difficulties in boarding with children, and delays. Further reasons related to car “*in front of the door*” (I1, 10) and comparably high fares discouraging people from switching to public transport (I1, I8, I15). The transport of (larger or heavier) objects or purchases was also one of the main reasons for car use. The experience of being subject to certain “*constraints*” that make the use of a car necessary in some cases, for example to ensure childcare or to go to work (I5, I9) was a reason as well.

*“And there (at the old office) I worked a lot of shifts, early, late and night, and if I had wanted to go to the early shift by public transport, I would have had to leave a DAY BEFORE. And then spending the night on the road might not have been so great.” (I9, 41)*.

Sometimes public transport and cycling was “*curiously*” problematic even within city districts because of “*super dangerous*” traffic conditions (I2, 14). The quality of cycling paths was sometimes described as “*catastrophic*” (I15, 72) and the behavior of other road users was experienced as an imposition on cyclists (I5, I4, I2). This aspect was important, because many interviewees with children had to cover short distances for everyday activities (e.g., to go shopping, to use the health care system, or to go to school or to the kindergarten). They were often unable to find infrastructural conditions allowing safe trips:

*“The cars drive like maniacs, they don’t take it into consideration and I just tried cycling there with both children and I really noticed that my heart couldn’t cope. (laughter) No bicycle lanes, extreme traffic jams, people are extremely annoyed and aggressive, there were times when I thought, wow, if I had a car, I would ACTUALLY drive this mini-mini-way. Simply for safety reasons for my children.” (I2, 14)*.

The narratives of two interview partners suggested that driving was a generational issue (I1, I7). For example, one interviewee reported that she was made fun of at her grandmother’s coffee party in the 1990s because she would rather have a bicycle with a trailer than a car. At the same time, the constant availability of cars from a young age onwards made it difficult to organize daily activities without the spontaneity that cars enable.

## Discussion

The aim of this study was to describe the characteristics and environmental values of people who participate voluntarily in local mobility interventions. The study aims also to give insights into the motivation to change individual mobility, the motivation for participating in a mobility project, persepctives on car replacement as well as reasons for car use. The data shows that participants are middle-aged, with good education levels, have a good subjective health status, show a rather medium environmental awareness and feel somewhat connected to nature. The consideration to live without the car was mainly present before the intervention. Participants felt motivated to take part because of incentives (money and discounts), commitment and to consolidate their considerations.

The combination of a high socioeconomic status and a rather high environmental attitude (cognitive dimension of environmental awareness) with the support of the qualitative statements revealing that most of the participants already wanted to leave their car before the intervention and the voluntary character of application to the intervention shows a high interest in the topic and an advanced motivation. This replicates a known phenomenon of interventions which can be referred to as recruiting bias or ‘preaching to the converted’ [38]. This means that effects of interventions are rather small because the participants are already living quite sustainable [39]. A Finish study with the same aim as the Bielefeld intervention also concluded that most of the participants considered a life without car already before the experiment. They also state that the longer this consideration has been going on, the easier was the shift [40]. Nevertheless, as in our case, these interventions can be an impetus to get rid of the car in the long term. The intervention phase could be used as an experimental phase for living without a car. However, as this is a known phenomenon (municipal) intervention planners need to find ways of recruiting to adress also not yet motivated persons for a more sustainable living.

Engaging in pro-environmental behavior shows widely socioeconomic differences. Hudde [41], for example, pointed out that people with higher education were more likely to cycle short distances than people with a lower education level, after controlling for gender and age. Additionally, the German Environmental Awareness Study analyzed the factors associated with alternative mobility (i.e., using bicycle, public transport or walking). It revealed that a higher education, age, pro-climatic values, living in an urban environment, and sufficient alternatives (subjectively perceived) are positively associated with more sustainable mobility [42]. Participants of our study mainly live in districts close to the city center which is in line with the results of Stieß et al. [42], which indicated that the urban environment offers better alternative infrastructure and thus facilitates mobility without car. Furthermore, Hudde [43] pointed out that the cycling boom in the last 20 years in Germany was mainly attributed to populations with higher education who live in medium sized and larger cities. These populations were three times more likely to use the bicycle than people with lower education living in rural areas. Overall, the impact of socio-demographic factors is, however, inconclusive [44, 45], as various studies reported a negative impact of high income on cycling behavior [46, 47]. Moreover, Parkin et al. [48] reported that low income was associated with less bicycle commuting as well.

Although participants in this study were already considering other ways of mobility before the intervention and showed rather low values in the perceived stress scale, they faced stressful situations at the beginning of the intervention. Those arose mainly from the way everyday life is organised. In our study 40% of the participants lived with children under the age of 12 years and interviewed families stated, that they often used the car to transport their children and to coordinate their leisure activities. An Australian study that analysed reasons for private car use with children found that most parents mentioned time constraints and weather [49]. Therefore, it can be assumed that switching to a life without a car initially leads to an increase in perceived stress. Especially, families with children below the age of 4 years tend to use their private car more. McCarthy et al. [50] analyzed which factors influence private car use or alternative transportation in families with children below 4 years in their review. They identified structural factors, psychosocial factors, household characteristics, and features of young children’s travel. In addition, participants with children stated that they often use the car to transport their children and coordinate their leisure activities. Similar results were reported in the cross-sectional study of bicycle commuting in six small U.S. cities [51]. They found that the participants with children used their trips to or from work for various errands, such as bringing and picking up their children from or to kindergarten or school or shopping for groceries. As a result, commuting by bike became much more difficult for them.

The lack of public transport connections and the traffic conditions were also experienced as stressful by the interviewees and reinforced car use. At the same time, participation in the intervention and the associated renunciation of car use made most participants to slow down a bit. Time spent waiting for buses, trams, and trains as well as time spent in those means of transportation were perceived as a welcome break. While participants had only a medium level of environmental awareness and rarely mentioned sustainability as the motivation for participation, some discovered that cycling promoted special experiences in and of nature. Whereas nature discovery was a consequence of cycling for participants in the present study, Semenescu and Coca [52] found that biospheric values, such as respecting earth, unity with nature, or protecting the environment [53] were strong predictors for cycling and reduced car use.

Interestingly, participants in our study tended to see themselves quite connected to nature. This result is in line with the results of two meta-analyses [20, 21] which showed, that people who are more connected to nature reported greater engagement in pro-environmental behavior. However, the single item scale (inclusion of nature in self) we used has one of the lowest associations with pro-environmental behavior [20]. This might explain why sustainability was hardly mentioned as a motivational aspect by participants.

That sustainability was not the predominant motive of mobility change could also relate to the low environmental awareness in the study population compared to the German Environmental Awareness study population. The participants of this study were less emotionally involved in environmental issues, were dealing rationally equal with environmental topics, and displayed more pro-environmental behavior than the representative German sample of the Environmental Awareness Study [42]. Ramos et al. [54] also showed in a European comparison that sustainability was not the most important motive for car sharing. They found that the convenience to have a car only when one needs it and the absence of maintenance responsibilites were more important reasons.

Andersson [55] found that climate morality is the most important factor for the motivation whether to use the private car. Climate morality is defined as feeling morally obligated to reduce the own greenhouse gas emissions. This is not directly comparable to the latent construct of environmental awareness but can be seen as an emotional way of dealing with an environmental issue, namely climate change. However, this result contradicts the findings of the current study as the participants here score rather low on the affective dimension towards environmental issues and are motivated to leave their cars anyway, at least for a minimum of three months. In this specific group the socioeconomic factors and cognitive dealing with environmental issues might be a better predictor for the motivation to reduce car use than emotional dealing.

Additionally, participants stated in the qualitative interviews that their motivation to use more alternative transportation was already present before the intervention took place. They rather used the project, with its different intervention strategies (e.g., incentives, group meetings, information events, and feedback), to turn motivation into action. According to the transtheoretical model (or stages of change model) developd by Prochaska and DiClemente [56] the participants in the study were in the stages contemplation (stage 2), because they intended to start the behavior in the foreseeable future and determination (stage 3), because the participants are ready to take action within the project. As stated by Vlaev et al. [57] combining incentives can show promising results for changing (environmental) behavior. It is further argued that mobility is more habitual than reflected behavior and therefore needs more triggers than only self-induced motivation. Habits are more easily changed by “key events”, like workplace or residence changes and interventions, than only through values [58].

A similar intervention project was piloted in the UK. Ten participants were selected in different cities (i.e., Birmingham, Bristol, Leeds, and London) to do without their car for one month. In the interviews participants stated similar concerns and motives for switching their mode of transportation. Similar to our study population, their biggest concern was the organization of family activities [59]. They also analyzed the carbon emission reduction of the alternative transport used which should be considered for all future interventions trying to reduce carbon emissions.

In our study, the participants frequently stated that, in addition to the distance to work, cycling was sometimes problematic due to unsafe traffic conditions. Both were major reasons that interviewees preferred commuting to work using the car. Other studies have come to similar conclusions. For example, Handy and Xing [51] showed that a short distance to work was a reason for commuting to work by bicycle. The study of Kruijf et al. [60] identified that the distance (less than 5 km increased the probability for using the bicycle) and time limitations were determinants of cycling to work. Ek et al. [61], who examined the motives of participants to walk or cycle when commuting, found that, besides the distance to work or school, the availability of safe routes were important for the choice to walk or cycle. Handy and Xing [51] also reported that both the short distance to work and safe bicycling infrastructure were predictors of commuting to work by bicycle. Blitz et al. [5] furthermore showed, that road conflicts reduced bicycle use, whereas the implementation of safe cycling routes increased bicycle use. This shows that individual behavior is also influenced by the (infra-)structural context.

### Limitations

The present study has some limitations worth noting. The sample size was comparatively small and did not allow statistical comparisons between subgroups. With a larger sample size inferential statistics could have been utilised. It should furthermore be kept in mind that the present results cannot be generalized to the entire Bielefeld or German population when interpreting the study because participants self-selected whether to parttake and are hence not representative of the overall population. In addition, it is possible that the people taking part in this study had a higher affinity for the topic of sustainable mobility and were more willing to give up the car than a random sample would have (selection bias). The analysis was moreover based on self-reported data, which means a possible response bias cannot be ruled out. Nevertheless, the results reported here appear to lead to similar conclusions as studies with larger sample sizes investigating the topic. The inclusion of control groups could elucidate the characteristics of people voluntarily participating in mobility interventions, their car use reasons, and motivation for participating in the intervention in realistic circumstances further.

## Conclusions

Urban projects can help to strengthen sustainability and Public Health by reaching the general population or specific populations like individual neighbourhoods, young people or commuters. In order to address different population groups in terms of age, gender, and socio-economic status, it is necessary to choose fitted recruitment strategies. Projects located at the structural (municipal) level, can provide essential information about the setting and individual behaviours that are necessary for sustainable urban development. The associated positive health effects, like stress reduction, should be given greater consideration. This requires transdisciplinary research and practice. Further studies should investigate long-term effects of mobility projects in different population subgroups.

### Electronic supplementary material

Below is the link to the electronic supplementary material.

Supplementary Material 1: Baseline questionnaire “3 months withoutthe car”.

Supplementary Material 2: Interview guide.

Supplementary Material 3: Overview of major and minor themes including definitions and quotes.

## Data Availability

The datasets generated and analysed during the current study are not publicly available due to data protection regulations in Germany but are available from the corresponding author on reasonable request.

## Abbreviations

WBGU: Wissenschaftlicher Beirat der Bundesregierung Globale Umweltveränderungen/ German Advisory Council on Global Change
CO_2_: Carbon dioxide
WHO: World Health Organization
INS: Extended Inclusion of Nature in Self scale
PSS: Perceived Stress Scale
SD: Standard deviation
U.S.: United States
