# Supplementary material for: 3 Months without the car in Bielefeld, Germany– a mixed-method study exploring individual motivation to participate in a municipal intervention
Source: BMC Public Health. 2024 Mar 11;24:759. doi: 10.1186/s12889-024-18266-7 (PMC10929177; doi:10.1186/s12889-024-18266-7)
Supplement: Supplementary file 1 — Supplementary Material 1: Baseline questionnaire “3 months withoutthe car”. [file 12889_2024_18266_MOESM1_ESM.pdf]

Baseline questionnaire “3 months without the car”

1. In which district/quarter of Bielefeld do you live?
    - Mitte
    - Heepen
    - Stieghorst
    - Brackwede
    - Gadderbaum
    - Dornberg
    - Senne
    - Sennestadt
    - Jöllenbeck
    - Schildesche

2. Habits in mobility
   First of all we want to get to know more about your mobility habits.

   How many ways did you do on Tuesday, 29th of March 2023? One way is defined as moving from one place to another by foot or means of transport. Outward and return journey count as two ways.

   Example: You go by bike to the daycare (way 1), then to the bakery (way 2), next is your workp place (way 3), back to the day care (way 4) and back home (way 5).

   Please type your answer here: [number]____

   If you did not leave the house please type 0.

3. [If answer in 2. was > 0] How many of those ways were connected ways?
   Example: You go to work and take your children to the daycare centre on the way.

   Please type your answer here: [number]____

4. [If answer in 2. was 0] You stated that you did not leave the house on the 29th of March 2023. Would you please explain shortly?

   Please type your answer here: [text]____

5. Did you use different means of transport this day?
    - Yes
    - No

6. [If answer in 5. was ‘Yes’] Which means of transport did you use?
    - Car

- Bike/E-Bike
- Bus
- Tram
- Train
- Scooter/E-Scooter
- Roller/E-Roller
- Others: [text]__________________________

7. Does your household currently have transport alternatives to the car?
    - Yes
    - No

8. [If answer in 7. was ‘Yes’] Which alternatives does your household currently have?
    - Bike
    - E-Bike
    - Cargo Bike
    - E-Cargo Bike
    - Scooter
    - E-Scooter
    - Roller
    - E-Roller
    - Others: [text]____

9. Do you currently have season ticket for public transport (bus, train, tram)?
    - Yes
    - No

10. [If answer in 9. was ‘Yes’] Which season ticket for public transport do you have?
    - Annual ticket
    - Monthly or weekly ticket
    - Tryout ticket for 3 months
    - BahnCard *(discount for train tickets)*
    - Others: [text]____

11. Do you currently have a membership for renting cars, e-scooter, etc.?
    - Yes
    - No

12. [If answer in 11. was ‘Yes’] Which membership do you have?
    - Car-Sharing membership
    - E-Scooter membership
    - Others: [text]____

13. Please choose now a route (e.g., to work, to your place of education, to relatives) that you travel regularly and several times a week.

    Please write down your chosen route here: [text]_______________________________

    When answering the following questions, please refer to the journey just indicated.

14. Which means of transportation do you currently use for the chosen journey?

    Please indicate for the following means of transportation how often do you currently use them for the chosen journey (1 “very often” to 6 “never”).

	1	2	3	4	5	6
car						
bike/e-bike/rental bike						
cargo bike/e-cargo bike/rental e-cargo bike						
scooter/e-scooter/ rental e-scooter						
Roller/rental e-roller						
bus						
tram						
car-sharing						
train						
collective call cab						
on-demand bus						

15. Are there other means of transport that you use very often or often for the chosen journey?

    [text]____

16. How important are the following criteria when choosing the means of transportation for your chosen journey?

    Please indicate for the following criteria how important do you think they are for choosing the means of transportation for your chosen journey (1 “very important” to 6 “not important at all”).

	1	2	3	4	5	6
flexibility						
promptness						
punctuality						
reliability						
traffic safety						
Protection against crime (assaults, theft)						
costs (e.g. ticket price)						
relaxation						
environmental compatibility						
convenience						

transportation of heavy or unwieldy objects						
reachability						
transfer frequency						
schedules						
operating hours						
comfort						

17. Are there other criteria that are very important or important to you which were not mentioned in the question above?

    [text]____

18. How satisfied are you with the following characteristics of public transportation?

    Please indicate how satisfied you are with the following characteristics of public transport (1 “very satisfied” to 6 “not satisfied at all”).

	1	2	3	4	5	6
flexibility						
promptness						
punctuality						
reliability						
traffic safety						
protection against crime (assaults, theft)						
costs (e.g. ticket price)						
relaxation						
environmental compatibility						
convenience						
transportation of heavy or unwieldy objects						
reachability						
transfer frequency						
schedules						
operating hours						
comfort						
suitability for everyday use						

19. Which characteristics of public transport are additionaly important?

    [text]____

20. How satisfied are you with the following characteristics of renting memberships?

    Please indicate how satisfied you are with the following characteristics of renting memberships (1 “very satisfied” to 6 “not satisfied at all”).

	1	2	3	4	5	6
reachability						
reliability						
availability						

functionality
selection option
comfort
costs (e.g. ticket price)
cleanliness
service

21. Which characteristics of renting memeberships are additionaly important?

    [text]____

22. Do children below the age of 12 years live in your household?
    - Yes
    - No

23. [If answer in 22. was 'Yes'] How many children below the age of 12 years live in your household?

    [number]____

24. How do you bring your children to, e.g. daycare centre, school, friends, leisure activities?

    Please indicate for the following means of transportation how often do you currently use them to bring your children to, e.g. daycare centre, school, friends, leisure activities (1 "very often" to 6 "never").

	1	2	3	4	5	6
car						
bike/e-bike/rental bike						
cargo bike/e-cargo bike/rental e-cargo bike						
scooter/e-scooter/ rental e-scooter						
Roller/rental e-roller						
bus						
tram						
car-sharing						
train						
collective call cab						
on-demand bus						

25. Are there other means of transport that you use very often or often to bring your children to, e.g. daycare centre, school, friends, leisure activities?

    [text]____

26. What is generally important to you when using public transport (bus, tram) in Bielefeld?

Please indicate for the following characteristics how important do you think they are for the public transport in Bielefeld (1 “very important” to 6 “not important at all”).

	1	2	3	4	5	6
many park-and-ride spaces						
close intervals of departure times						
expansion of the tram routes						
low prices						
clean buses and trams						
clean stops and platforms						
lots of security						
reliabilty						

27. Are there additional characteristics that you think are important for the public transport in Bielefeld that were not mentioned in the question above?

    [text]____

28. How important are the following characteristics for you when cycling?

    Please indicate for the following characteristics of cycling how important they are to you (1 "very important" to 6 "not important at all ").

	1	2	3	4	5	6
many cycle paths						
safe cycle paths						
bike-friendly traffic lights						
plenty of parking options for the bike						
safe parking options for the bike						
consideration by car drivers						
consideration by other cyclists						

29. Are there additional characteristics that are important for you when cycling that were not mentioned in the question above?

    [text]____

Well-being and health

In the following section we want to know more about your well-being and health.

30. Please indicate for each of the five statements which is closest to how you have been feeling over the last two weeks.

In the last 2 weeks…

	All of the time	Most of the time	More than half the time	Less than half the time	Some of the time	At no time
I have felt cheerful in good spirits.						
I have felt calm and relaxed.						

I have felt active and vigorous.

I woke up feeling fresh and rested.

My daily life has been filled with things that interest me.

31. The following question is regarding your health. Please indicate which statement represents best your general health.
    - Very good
    - Good
    - Mediocre
    - Bad
    - Very bad

32. The questions in this scale ask you about your feelings and thoughts during the last month. In each case, you will be asked to indicate how often you felt or thought a certain way.

    If you think about the last month…

	Never	Almost never	Sometimes	Quite often	Very often
… how often have you been upset because of something that happened unexpectedly?					
… how often have you felt that you were unable to control the important things in your life?					
… how often have you felt nervous and ‘‘stressed’’?					
… how often have you felt confident about your ability to handle your personal problems?					
… how often have you felt that things were going your way?					
… how often have you found that you could not cope with all the things that you had to do?					
… how often have you been able to control irritations in your life?					
… how often have you felt that you were on top of things?					
… how often have you been angered because of things that were outside of your control?					
… how often have you felt difficulties were piling up so high that you could not overcome them?					

Nature and environment

In the following section we want to get to know more about you and your relation to nature and the environment.

33. Please choose one of the following pictures which describes best your relation to the natural environment.

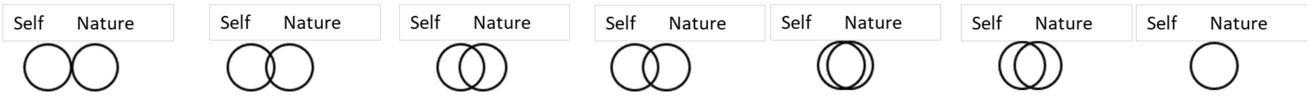

34. To what extent do you agree with the following statements?

	Do not agree at all	Do not agree	Partially agree	Do agree	Totally agree
There are natural limits to growth that our industrialised world has long since reached.					
In favour of the environment, we should all be prepared to reduce our current standard of living.					
It worries me when I think about the environmental conditions in which future generations will probably have to live in.					
Climate change also threatens the basis of life here in Germany.					
Man-made environmental problems such as deforestation or plastic in the oceans outrage me.					
Other things are important for a good life than the environment and nature.					
Each and every one of us is responsible for ensuring that we leave following generations an environment worth living in.					
The environmental problem is greatly exaggerated by many environmentalists.					
I am pleased about initiatives that simply try out sustainable lifestyles (e.g. ecovillages, the SlowFood movement).					

We should not consume more resources than can be regrown.

It makes me angry when I see how Germany is missing its climate protection targets.

More environmental protection also means better quality of life and health for everyone.

We need more economic growth in the future, even if this has a negative impact on the environment.

I get annoyed when environmentalists want to tell me how I should live.

We have to find ways to live well independently of economic growth.

35. Please think about your everyday life and indicate how often you do the following actions. Chose from “Never” to “Always”.

	Never	Rarely	Sometimes	Often	Always
I buy food from certified organic farming.					
When shopping, I choose products with an environmental label (e.g. Blue Angel, EU organic label or EU Ecolabel).					
I eat meat at the main meals.					
For my everyday journeys, I use my bike, public transport or walk.					
When buying household appliances, I choose particularly energy-efficient appliances (A+++ or A++ energy efficiency label).					
I donate money to environmental or nature conservation groups.					
I am actively involved in environmental protection and nature conservation.					

36. My household purchases..
    - Conventional electricity
    - Green electricity

Almost done! Finally, we would like to know something about you personally.

37. Which gender do you feel you belong to?
    - Female
    - Male
    - Diverse

38. Please write your age in years here: [number]_____________________________

39. How many persons live in your household?

    [number]____

40. What is your highest achieved educational level?
    - No degree
    - Lower secondary degree
    - Higher secondary degree
    - A-Levels
    - Degree from a university
    - Other degree
