# Supplementary material for: 3 Months without the car in Bielefeld, Germany– a mixed-method study exploring individual motivation to participate in a municipal intervention
Source: BMC Public Health. 2024 Mar 11;24:759. doi: 10.1186/s12889-024-18266-7 (PMC10929177; doi:10.1186/s12889-024-18266-7)
Supplement: Supplementary file 2 — Supplementary Material 2: Interview guide. [file 12889_2024_18266_MOESM2_ESM.pdf]

Interview guide '3 months without car' (t_0)

1. If you think of a normal weekday: what do you have to do on this day?
    a. Please describe your daily routine.
    b. How do you travel?
2. What experiences have you had with the means of transport mentioned?
    a. Why have you chosen this particular mode of transport?
    b. Why haven't you chosen the bus/train/bike/car sharing?
    c. In which situations do you use public transport / bike?
    d. What experiences have you had with public transport so far?
    e. What challenges do you face when travelling by bike or walking?
    f. What factors influence your choice of transport at the moment?
3. Why did you decide to take part in the project?
    a. What do you hope to gain from the participation?
    b. What challenges do you face? How do you intend to overcome these challenges?
    c. What do you need for achieving your goals?
