# Supplementary material for: 3 Months without the car in Bielefeld, Germany– a mixed-method study exploring individual motivation to participate in a municipal intervention
Source: BMC Public Health. 2024 Mar 11;24:759. doi: 10.1186/s12889-024-18266-7 (PMC10929177; doi:10.1186/s12889-024-18266-7)
Supplement: Supplementary file 3 — Supplementary Material 3: Overview of major and minor themes including definitions and quotes. [file 12889_2024_18266_MOESM3_ESM.pdf]

Table S1: Overview of major and minor themes including definitions and quotes

Code	Subcode	Definition	Example Quote
**Motivation for participation** Motivation for participation due to inherent reasons of the intervention	Financial incentives	Importance of financial incentives for project participation and motivation to switch	*I also want to reduce my living costs, of course (...). One incentive is of course that you get 400€ and can EXPERIENCE it all* (I10, 97)
	Commitment	Commitment as an important factor in encouraging participation in the project over the entire period.	*Because we also want to do without it beforehand [...], and if you are so committed or a bit, well [...] have committed ourselves, yes, but then it's more binding, isn't it?* (I6, 74)
	Trying out alternatives	Trying out alternatives as part of the intervention, which can lead to a change in car use in the long term.	*This is now the opportunity to try out whether you really want to do without it [the car] completely.* (I1, 29)
	Life course experiences	Intervention as a corrective for the positive experiences of car use in the course of life.	*I think it's more of a feeling thing. I grew up in a village and got my first car when I was 18. I saved up money for it for a long time and then the world of mobility was the car* (I11, 41)
	Sustainable living	Reasons for sustainable mobility	*Yes, and in principle it's also the case that we as a family are also looking at how we can live even more sustainably or environmentally consciously* (I6, 186f.)
**Motivation to change** Personal motivation and experiences for being part of the intervention and behaviour change	Advantages of cycling	Advantages of using a bicycle in daily life	*It takes me half an hour by car. And the difference to the car is that it ALWAYS takes me 50 minutes by bike (...), but it doesn't happen to me that the tunnel is closed or that there are*

			roadworks or that there is dew (I8, 44ff.)
	Disadvantage of car use	Disadvantages of a car and car use that were experienced before the intervention	*Because finding a parking space here in the city is just so annoying* (I8, 27)
	Strategies for dealing with challenges	Strategies prepared in advance of the intervention that make it easier to start living without a car.	*Oh, I had to rethink my route to work and I'm still trying out different routes because I have the ABSOLUTE car coding in my head. For a start, I cycled the way I would in my car, but as a cyclist I don't HAVE to.* (I6, 104)
	Little changes	Descriptions of few to no changes as a result of the project, because the car had already been dispensed in the run-up to the intervention.	*To be honest, I don't think much or nothing has changed for me, because I just/ I just cycle everywhere* (I12, 3)
**Perspectives on car replacement** Experiences of the switch to public transport, car-sharing and/or cycling	Discovering	New insights that could be gained on alternative forms of mobility through the project	*But it does revolve a bit around these thoughts of what I want to try out and that's actually my goal in this phase, to really test as many things as possible that I haven't even considered yet and that I haven't dealt with* (I10, 12)
	Get into it	Descriptions of the challenges in dealing with new ways and means of transport	*So, we would also have to get to grips with car sharing again* (I6, 95)
	Unreachable destinations	Statements on the challenges of reaching poorly connected places	*My brother lives in the countryside. (...) I looked into how I could get there. I actually COULD get there, because he doesn't live in a place that's particularly inconvenient for public transport (...). But I have to get away again.* (I10, Z. 64)

	Traffic conditions	Description of experiences as non-motorised road users	*(...) That's why I drive on the road, which is allowed, and I don't know how often I get honked at and cut off (...) You really have to have a thick skin* (I8, lines 65ff.)
**Reasons for car use**<br>Reasons and advantages of car use	Time flexibility	Descriptions on the temporal advantages of using a car	*Because just getting into the car, driving somewhere, picking something up, doing something else, that was always the case* (I6, 21)
	Freedom	Situations in which the interviewees emphasise the flexibility of using the car and describe feelings of freedom	*I travel a lot and pack my appointments tightly and yes, that's not possible now* (I7, 353)
	Organisation of working life	The factors in favour of using a car and against using a bike when organising work commitments	*I always wanted to do this [use a bike] anyway, but somehow, then the time was too short and then I was able to use the journeys again to get to work THEN* (I6, 7)
	Weather	Factors that can result from the weather as a natural influencing factor	*The only thing that is really a huge factor is always the weather. You have to be able to cope with that* (I8, 253)
	Travel distance	Individual reasons for using a car that are inherent in the distance that need to be travelled	*Yes, but then to the DIY store, to [neighbourhood], that's a bit too far for me by bike* (I6, 92)
	Habitual behaviour	Car use as a routine and natural behaviour pattern in certain situations	*Well, I'm a habitual car driver to work, even though it's actually only five kilometres* (I6, 4)
	Organisation of everyday life	Situations in which car use seems indispensable in everyday life.	*It didn't used to be a problem, but now it's like that, because of all the children's appointments, such a construct has built up that it has to work. If just one cogwheel is somehow loose or doesn't work*

			properly (makes the sound of something collapsing) (I9, 72)
